# Supplementary material for: Modifiable risk factors and inflammation-related proteins in polymyalgia rheumatica: genome-wide meta-analysis and Mendelian randomization
Source: Rheumatology (Oxford). 2024 May 24;64(5):3012–8. doi: 10.1093/rheumatology/keae308 (PMC7616751; doi:10.1093/rheumatology/keae308)
Supplement: keae308_Supplementary_Data [file keae308_supplementary_data.docx]

Supplementary materials for:

**Modifiable risk factors and inflammation-related proteins in polymyalgia rheumatica: genome-wide meta-analysis and Mendelian randomisation**

Sizheng Steven Zhao^1^, Sarah L Mackie^2,3^, Susanna C Larsson^4,5^, Stephen Burgess^6,7,8^, Shuai Yuan^4^

1 Centre for Musculoskeletal Research, Division of Musculoskeletal and Dermatological Science, School of Biological Sciences, Faculty of Biological Medicine and Health, The University of Manchester, Manchester Academic Health Science Centre, Manchester, UK

2 Leeds Institute of Rheumatic and Musculoskeletal Medicine, University of Leeds, Leeds, UK

3 National Institute for Health Research Leeds Biomedical Research Centre, Leeds Teaching Hospitals, University of Leeds, Leeds, UK

4 Unit of Cardiovascular and Nutritional Epidemiology, Institute of Environmental Medicine, Karolinska Institutet, Nobelsväg 13, 17177, Stockholm, Sweden

5 Department of Surgical Sciences, Uppsala University, Dag Hammarskjölds Väg 14B, 75185, Uppsala, Sweden

6 British Heart Foundation Cardiovascular Epidemiology Unit, Department of Public Health and Primary Care, University of Cambridge, Cambridge, UK

7 Heart and Lung Research Institute, University of Cambridge, Cambridge UK

8 Medical Research Council Biostatistics Unit, University of Cambridge, Cambridge, UK

Contents

[Table S1. Details for genome-wide association studies from which data were obtained. 2](#_Toc164585079)

[Table S2. Genetic associations from the meta-analysis and individual populations. 4](#_Toc164585080)

[Table S3. Mendelian randomization estimates for the association between risk factors and polymyalgia rheumatica. 5](#_Toc164585081)

[Table S4. Mendelian randomization estimates for the association between inflammatory markers and polymyalgia rheumatica. 6](#_Toc164585082)

.

# Table S1. Details for genome-wide association studies from which data were obtained.

| Exposure | No. SNPs | *F* statistic | Unit | PubMed ID | Sample size | Ancestry | Measurement |
| --- | --- | --- | --- | --- | --- | --- | --- |
| Body mass index | 311 | 77.2 | SD | 30239722 | 806,834 | European | Calculated as weight (kg) divided by height (m) squared. Weight and height data were measured (most studies) or self-reported. |
| Waist circumference | 573 | 55.8 | SD | 30239722 | 697,734 | European | Measured (most studies) or self- reported. |
| Visceral adiposity | 294 | 50.2 | SD | 31501611 | 397,170 | European | Measured by DXA among 5,109 participants and then predicted among 502,638 using the calibrated prediction models. |
| Smoking initiation | 313 | 40.0 | SD in prevalence of smoking initiation | 36477530 | 2,669,029 | European | Smoking behaviours were measured by questionnaires. Smoking initiation is a binary phenotype representing whether an individual had ever smoked cigarettes regularly in their life. |
| Smoking intensity (cigarettes/day) | 65 | 110.8 | SD in number of cigarettes per day | 36477530 | 618,489 | European | Self-reported average number of cigarettes per day among current and former smokers. |
| Alcohol consumption | 114 | 81.0 | SD increase of log- transformed alcoholic drinks/wk | 36477530 | 2,428,851 | European | Self-reported by questionnaires. |
| Coffee consumption | 12 | 196.1 | 50% change | 31046077 | 375,833 | European | Assessed by a 24-h recall questionnaire. |
| Moderate to vigorous physical activity | 16 | 40.6 | Being active vs. inactive | 36071172 | 298,506 | European | Measured by questionnaires and dichotomized due to the zero- inflated negative binomial nature of the distribution. |
| Inflammatory markers | 1-19 | ≥29.6 | SD | 37205426 | 876-55,792 | European | Measured by BioRad BioPlex assays, SomaScan, or Olink platforms. |

SD, standard deviation; SNPs, single nucleotide polymorphisms.

# Table S2. Genetic associations from the meta-analysis and individual populations.

|  |  | Meta-analysis | | | | | FinnGen | | | | UKB | | | |
| --- | --- | --- | --- | --- | --- | --- | --- | --- | --- | --- | --- | --- | --- | --- |
| SNP | **Chromosome: position** | **EA/OA** | **Beta** | **SE** | **p** | **p_het** | **EAF** | **Beta** | **SE** | **P** | **EAF** | **Beta** | **SE** | **P** |
| rs11694915 | 2:102710068 | A/T | -0.098 | 0.017 | 1.40E-08 | 0.954 | 0.433 | -0.099 | 0.023 | 2.44E-05 | 0.331 | -0.097 | 0.026 | 1.48E-04 |
| rs748741 | 9:127089775 | A/G | -0.096 | 0.017 | 2.31E-08 | 0.694 | 0.379 | 0.102 | 0.024 | 1.49E-05 | 0.388 | 0.089 | 0.025 | 3.77E-04 |
| rs615540 | 11:64123838 | A/G | 0.106 | 0.017 | 3.70E-10 | 0.171 | 0.471 | -0.084 | 0.023 | 3.13E-04 | 0.429 | -0.130 | 0.025 | 1.12E-07 |
| rs2760985 | 6:32566398 | A/G | 0.531 | 0.021 | 7.78E-142 | 0.0001 | 0.150 | 0.453 | 0.029 | 3.83E-54 | 0.290 | 0.613 | 0.030 | 7.85E-93 |
| rs7731626 | 5:55444683 | A/G | -0.162 | 0.018 | 4.59E-19 | 0.825 | 0.279 | -0.166 | 0.026 | 2.95E-10 | 0.342 | -0.158 | 0.025 | 2.61E-10 |

EA, effect allele; OA, other allele; SE standard error; p_het, p-value for heterogeneity; EAF, effect allele frequency; UKB, UK Biobank.

# Table S3. Mendelian randomization estimates for the association between risk factors and polymyalgia rheumatica.

|  |  | IVW-fixed | | | IVW-random | | | | Weighted median | | | | MR-Egger | | | | | |
| --- | --- | --- | --- | --- | --- | --- | --- | --- | --- | --- | --- | --- | --- | --- | --- | --- | --- | --- |
| Exposure | **No. SNP** | **beta** | **se** | **p** | **beta** | **se** | **p** | **beta** | | **se** | **p** | **beta** | | **se** | **p** | **intercept** | **intercept p** |  |
| Body mass index | 311 | 0.149 | 0.060 | 0.013 | 0.149 | 0.067 | 0.025 | 0.142 | | 0.116 | 0.221 | 0.251 | | 0.164 | 0.127 | -0.002 | 0.499 |  |
| Waist circumference | 43 | 0.123 | 0.096 | 0.202 | 0.123 | 0.121 | 0.311 | 0.252 | | 0.163 | 0.121 | 0.590 | | 0.324 | 0.076 | -0.015 | 0.128 |  |
| Visceral adiposity | 294 | 0.202 | 0.056 | 0.0003 | 0.202 | 0.063 | 0.001 | 0.223 | | 0.103 | 0.030 | 0.181 | | 0.200 | 0.368 | 0.000 | 0.910 |  |
| Smoking initiation | 346 | 0.105 | 0.085 | 0.218 | 0.105 | 0.099 | 0.288 | 0.117 | | 0.131 | 0.371 | 0.925 | | 0.405 | 0.023 | -0.010 | 0.037 |  |
| Cigarettes per day | 65 | 0.274 | 0.099 | 0.006 | 0.274 | 0.104 | 0.008 | 0.198 | | 0.159 | 0.214 | 0.299 | | 0.192 | 0.125 | -0.001 | 0.878 |  |
| Alcohol consumption | 114 | -0.148 | 0.126 | 0.241 | -0.148 | 0.126 | 0.240 | -0.072 | | 0.226 | 0.751 | -0.359 | | 0.294 | 0.224 | 0.003 | 0.427 |  |
| Coffee consumption | 11 | 0.103 | 0.126 | 0.413 | 0.103 | 0.140 | 0.462 | 0.058 | | 0.160 | 0.717 | -0.138 | | 0.270 | 0.621 | 0.013 | 0.322 |  |
| Moderate to vigorous physical activity | 17 | -0.225 | 0.181 | 0.214 | -0.225 | 0.137 | 0.100 | -0.109 | | 0.243 | 0.655 | 0.370 | | 0.971 | 0.709 | -0.015 | 0.542 |  |

# Table S4. Mendelian randomization estimates for the association between inflammatory markers and polymyalgia rheumatica.

|  |  |  |  | IVW-fixed | | | IVW-random | | | Weighted median | | | MR-Egger | | | | |
| --- | --- | --- | --- | --- | --- | --- | --- | --- | --- | --- | --- | --- | --- | --- | --- | --- | --- |
| Exposure | **No. SNP** | **F stat** | **FDR** | **beta** | **se** | **p** | **beta** | **se** | **p** | **beta** | **se** | **p** | **beta** | **se** | **p** | **intercept** | **intercept p** |
| Interleukin-1 receptor-like 2 | 3 | 185.3 | 1.23E-30 | 0.226 | 0.063 | 3.09E-04 | 0.226 | 0.019 | 1.89E-32 | 0.233 | 0.066 | 4.31E-04 | 0.222 | 0.180 | 0.434 | 0.001 | 0.984 |
| Serum amyloid A-2 protein | 3 | 282.9 | 3.22E-08 | 0.054 | 0.044 | 2.26E-01 | 0.054 | 0.009 | 9.91E-10 | 0.052 | 0.046 | 2.51E-01 | 0.050 | 0.088 | 0.673 | 0.002 | 0.966 |
| C-X-C motif chemokine 6 | 4 | 403.3 | 1.05E-05 | 0.088 | 0.038 | 2.06E-02 | 0.088 | 0.018 | 4.85E-07 | 0.090 | 0.038 | 1.76E-02 | 0.542 | 0.570 | 0.443 | -0.188 | 0.509 |
| Interleukin-1 receptor type 2 | 2 | 684.7 | 5.96E-02 | 0.107 | 0.037 | 4.10E-03 | 0.107 | 0.136 | 4.29E-01 |  |  |  |  |  |  |  |  |
| Fractalkine | 1 | 345.7 | 5.96E-02 | 0.234 | 0.085 | 5.80E-03 |  |  |  |  |  |  |  |  |  |  |  |
| Interleukin-1 receptor type 1 | 2 | 78.4 | 5.96E-02 | -0.342 | 0.126 | 6.42E-03 | -0.342 | 0.293 | 2.44E-01 |  |  |  |  |  |  |  |  |
| Interleukin-1 receptor antagonist protein | 4 | 150.4 | 5.96E-02 | -0.188 | 0.085 | 2.64E-02 | -0.188 | 0.068 | 5.63E-03 | -0.219 | 0.093 | 1.92E-02 | -0.348 | 0.357 | 0.432 | 0.027 | 0.690 |
| C-type lectin domain family 11 member A | 1 | 67.4 | 1.19E-01 | 0.208 | 0.085 | 1.46E-02 |  |  |  |  |  |  |  |  |  |  |  |
| Interleukin-6 receptor subunit alpha | 11 | 617.7 | 2.11E-01 | -0.054 | 0.025 | 2.90E-02 | -0.054 | 0.025 | 3.25E-02 | -0.040 | 0.035 | 2.56E-01 | -0.115 | 0.099 | 0.274 | 0.026 | 0.540 |
| C-reactive protein | 1 | 202.1 | 2.11E-01 | 0.213 | 0.098 | 3.00E-02 |  |  |  |  |  |  |  |  |  |  |  |
| Pro-interleukin-16 | 5 | 253.0 | 2.47E-01 | -0.092 | 0.046 | 4.47E-02 | -0.092 | 0.045 | 4.18E-02 | -0.084 | 0.050 | 9.26E-02 | -0.032 | 0.061 | 0.641 | -0.022 | 0.238 |
| Interleukin-12 receptor subunit beta-2 | 1 | 92.5 | 2.50E-01 | -0.144 | 0.072 | 4.61E-02 |  |  |  |  |  |  |  |  |  |  |  |
| E-selectin | 1 | 47.1 | 4.00E-01 | -0.283 | 0.164 | 8.56E-02 |  |  |  |  |  |  |  |  |  |  |  |
| C-X-C motif chemokine 16 | 2 | 162.1 | 4.00E-01 | -0.168 | 0.098 | 8.62E-02 | -0.168 | 0.034 | 4.96E-07 |  |  |  |  |  |  |  |  |
| Placenta growth factor | 1 | 136.6 | 4.91E-01 | -0.230 | 0.145 | 1.13E-01 |  |  |  |  |  |  |  |  |  |  |  |
| Interleukin-1 receptor-like 1 | 17 | 357.2 | 4.94E-01 | 0.044 | 0.028 | 1.15E-01 | 0.044 | 0.028 | 1.22E-01 | 0.036 | 0.038 | 3.39E-01 | -0.014 | 0.113 | 0.904 | 0.026 | 0.602 |
| Interleukin-2 receptor subunit beta | 1 | 72.4 | 5.35E-01 | 0.246 | 0.170 | 1.48E-01 |  |  |  |  |  |  |  |  |  |  |  |
| C-C motif chemokine 7 | 1 | 263.1 | 5.35E-01 | -0.064 | 0.044 | 1.48E-01 |  |  |  |  |  |  |  |  |  |  |  |
| Interleukin-17D | 1 | 32.9 | 7.66E-01 | -0.087 | 0.073 | 2.36E-01 |  |  |  |  |  |  |  |  |  |  |  |
| C-C motif chemokine 4 | 5 | 86.9 | 7.66E-01 | -0.083 | 0.078 | 2.88E-01 | -0.083 | 0.069 | 2.26E-01 | -0.127 | 0.097 | 1.87E-01 | -0.230 | 0.144 | 0.209 | 0.038 | 0.312 |
| C-C motif chemokine 8 | 1 | 349.6 | 7.67E-01 | -0.021 | 0.019 | 2.74E-01 |  |  |  |  |  |  |  |  |  |  |  |
| Haptoglobin | 17 | 332.7 | 7.67E-01 | -0.021 | 0.019 | 2.77E-01 | -0.021 | 0.019 | 2.67E-01 | -0.010 | 0.026 | 7.12E-01 | 0.073 | 0.063 | 0.269 | -0.056 | 0.141 |
| C-C motif chemokine 16 | 1 | 2035.4 | 7.67E-01 | -0.023 | 0.021 | 2.83E-01 |  |  |  |  |  |  |  |  |  |  |  |
| Fibroblast growth factor 23 | 1 | 40.1 | 7.67E-01 | 0.247 | 0.239 | 3.02E-01 |  |  |  |  |  |  |  |  |  |  |  |
| Pro-epidermal growth factor | 1 | 53.9 | 7.67E-01 | -0.134 | 0.135 | 3.20E-01 |  |  |  |  |  |  |  |  |  |  |  |
| C-X-C motif chemokine 5 | 1 | 67.7 | 7.67E-01 | -0.091 | 0.092 | 3.25E-01 |  |  |  |  |  |  |  |  |  |  |  |
| Hepatocyte growth factor | 1 | 128.8 | 7.67E-01 | -0.164 | 0.168 | 3.30E-01 |  |  |  |  |  |  |  |  |  |  |  |
| Growth-regulated alpha protein | 5 | 148.2 | 7.67E-01 | -0.060 | 0.069 | 3.86E-01 | -0.060 | 0.060 | 3.18E-01 | -0.099 | 0.076 | 1.93E-01 | -0.215 | 0.115 | 0.158 | 0.050 | 0.190 |
| Eotaxin | 1 | 30.6 | 7.98E-01 | 0.206 | 0.224 | 3.56E-01 |  |  |  |  |  |  |  |  |  |  |  |
| Interleukin-2 receptor subunit alpha | 1 | 34.5 | 8.17E-01 | -0.247 | 0.280 | 3.77E-01 |  |  |  |  |  |  |  |  |  |  |  |
| Vascular endothelial growth factor A | 11 | 76.3 | 8.52E-01 | -0.053 | 0.058 | 3.57E-01 | -0.053 | 0.068 | 4.33E-01 | -0.065 | 0.080 | 4.15E-01 | 0.143 | 0.121 | 0.266 | -0.039 | 0.093 |
| Serum amyloid A-1 protein | 5 | 244.0 | 8.52E-01 | 0.034 | 0.039 | 3.80E-01 | 0.034 | 0.043 | 4.30E-01 | 0.049 | 0.041 | 2.27E-01 | 0.066 | 0.079 | 0.467 | -0.015 | 0.650 |
| C-C motif chemokine 27 | 1 | 88.7 | 8.52E-01 | -0.084 | 0.107 | 4.32E-01 |  |  |  |  |  |  |  |  |  |  |  |
| C-C motif chemokine 14 | 4 | 162.3 | 8.65E-01 | 0.027 | 0.027 | 3.03E-01 | 0.027 | 0.037 | 4.60E-01 | 0.032 | 0.030 | 2.83E-01 | 0.036 | 0.137 | 0.816 | -0.007 | 0.952 |
| C-C motif chemokine 3 | 3 | 236.5 | 8.65E-01 | 0.022 | 0.046 | 6.40E-01 | 0.022 | 0.030 | 4.66E-01 | 0.020 | 0.045 | 6.54E-01 | 0.025 | 0.076 | 0.798 | -0.001 | 0.963 |
| C-C motif chemokine 17 | 3 | 77.8 | 9.54E-01 | 0.048 | 0.057 | 4.01E-01 | 0.048 | 0.094 | 6.15E-01 | 0.068 | 0.070 | 3.32E-01 | 0.069 | 0.475 | 0.908 | -0.006 | 0.970 |
| C-C motif chemokine 22 | 3 | 50.6 | 9.54E-01 | -0.039 | 0.058 | 5.03E-01 | -0.039 | 0.090 | 6.66E-01 | 0.026 | 0.074 | 7.27E-01 | 0.150 | 0.142 | 0.481 | -0.062 | 0.369 |
| Interleukin-23 receptor | 2 | 66.2 | 9.54E-01 | 0.038 | 0.062 | 5.39E-01 | 0.038 | 0.060 | 5.24E-01 |  |  |  |  |  |  |  |  |
| C-C motif chemokine 2 | 1 | 54.2 | 9.54E-01 | 0.182 | 0.319 | 5.69E-01 |  |  |  |  |  |  |  |  |  |  |  |
| Serum amyloid P-component | 2 | 78.2 | 9.54E-01 | -0.024 | 0.049 | 6.19E-01 | -0.024 | 0.002 | 7.65E-46 |  |  |  |  |  |  |  |  |
| Interleukin-17 receptor A | 9 | 362.7 | 9.54E-01 | 0.012 | 0.027 | 6.47E-01 | 0.012 | 0.031 | 6.93E-01 | 0.015 | 0.032 | 6.32E-01 | -0.018 | 0.061 | 0.780 | 0.016 | 0.580 |
| Fibroblast growth factor 7 | 1 | 49.9 | 9.54E-01 | -0.041 | 0.097 | 6.70E-01 |  |  |  |  |  |  |  |  |  |  |  |
| Fibrinogen gamma chain | 2 | 75.4 | 9.54E-01 | 0.043 | 0.107 | 6.87E-01 | 0.043 | 0.145 | 7.66E-01 |  |  |  |  |  |  |  |  |
| C-C motif chemokine 20 | 1 | 61.0 | 9.54E-01 | -0.122 | 0.307 | 6.90E-01 |  |  |  |  |  |  |  |  |  |  |  |
| Antithrombin-III | 1 | 31.1 | 9.54E-01 | 0.109 | 0.279 | 6.97E-01 |  |  |  |  |  |  |  |  |  |  |  |
| Vascular endothelial growth factor C | 1 | 205.5 | 9.54E-01 | 0.010 | 0.026 | 6.99E-01 |  |  |  |  |  |  |  |  |  |  |  |
| Interleukin-8 | 1 | 58.5 | 9.54E-01 | -0.089 | 0.234 | 7.04E-01 |  |  |  |  |  |  |  |  |  |  |  |
| Pro-adrenomedullin | 4 | 53.3 | 9.54E-01 | 0.021 | 0.108 | 8.45E-01 | 0.021 | 0.044 | 6.34E-01 | 0.054 | 0.121 | 6.53E-01 | -0.093 | 0.422 | 0.846 | 0.013 | 0.806 |
| Plasminogen activator inhibitor 1 | 1 | 73.3 | 9.98E-01 | -0.062 | 0.199 | 7.55E-01 |  |  |  |  |  |  |  |  |  |  |  |
| Tumor necrosis factor ligand superfamily member 10 | 2 | 189.7 | 9.98E-01 | 0.025 | 0.088 | 7.73E-01 | 0.025 | 0.160 | 8.74E-01 |  |  |  |  |  |  |  |  |
| Prothrombin | 1 | 69.9 | 9.98E-01 | 0.033 | 0.170 | 8.45E-01 |  |  |  |  |  |  |  |  |  |  |  |
| Interleukin-27 receptor subunit alpha | 1 | 1093.4 | 9.98E-01 | 0.003 | 0.023 | 8.78E-01 |  |  |  |  |  |  |  |  |  |  |  |
| C-C motif chemokine 25 | 1 | 164.7 | 9.98E-01 | -0.004 | 0.029 | 8.92E-01 |  |  |  |  |  |  |  |  |  |  |  |
| C-C motif chemokine 5 | 1 | 67.5 | 9.98E-01 | -0.012 | 0.120 | 9.17E-01 |  |  |  |  |  |  |  |  |  |  |  |
| Macrophage colony-stimulating factor 1 | 1 | 116.9 | 9.98E-01 | -0.015 | 0.154 | 9.25E-01 |  |  |  |  |  |  |  |  |  |  |  |
| Interleukin-7 receptor subunit alpha | 1 | 191.5 | 9.98E-01 | -0.009 | 0.105 | 9.33E-01 |  |  |  |  |  |  |  |  |  |  |  |
| Interleukin-18 | 1 | 450.6 | 9.98E-01 | -0.007 | 0.086 | 9.34E-01 |  |  |  |  |  |  |  |  |  |  |  |
| C-X-C motif chemokine 10 | 1 | 36.4 | 9.98E-01 | 0.017 | 0.221 | 9.38E-01 |  |  |  |  |  |  |  |  |  |  |  |
| Macrophage migration inhibitory factor | 1 | 39.0 | 9.98E-01 | -0.007 | 0.111 | 9.49E-01 |  |  |  |  |  |  |  |  |  |  |  |
| C-X-C motif chemokine 11 | 1 | 84.6 | 9.98E-01 | -0.004 | 0.074 | 9.57E-01 |  |  |  |  |  |  |  |  |  |  |  |
| Intercellular adhesion molecule 1 | 3 | 86.3 | 9.98E-01 | -0.003 | 0.059 | 9.59E-01 | -0.003 | 0.051 | 9.53E-01 | 0.018 | 0.065 | 7.81E-01 | 0.084 | 0.094 | 0.535 | -0.026 | 0.447 |
| Mannose-binding protein C | 2 | 852.6 | 9.98E-01 | 0.001 | 0.020 | 9.64E-01 | 0.001 | 0.049 | 9.85E-01 |  |  |  |  |  |  |  |  |
| Interleukin-17 receptor D | 1 | 331.3 | 9.98E-01 | 0.001 | 0.057 | 9.85E-01 |  |  |  |  |  |  |  |  |  |  |  |
| C-X-C motif chemokine 9 | 1 | 46.7 | 9.98E-01 | -0.001 | 0.122 | 9.92E-01 |  |  |  |  |  |  |  |  |  |  |  |
| Interleukin-6 receptor subunit beta | 6 | 139.3 | 9.98E-01 | 0.000 | 0.051 | 9.98E-01 | 0.000 | 0.048 | 9.98E-01 | 0.002 | 0.057 | 9.74E-01 | 0.014 | 0.086 | 0.875 | -0.004 | 0.843 |
